# Gutta Percha

**Published:** 1848-07

**Authors:** 


From the Dental Recorder.

### GUTTA PERCHA.

This article bids fair to be as useful in the arts as caoutchouc has been. It has already been extensively used for soles of shoes, and is generally preferred to rubber on account of its superior firmness and solidity. It is an excellent material for bands for the Dentist's lathe, the ends may be cemented together, which causes it to run perfectly, still; neither is it affected in the least by the dryness or dampness of the atmosphere, as those are which are constructed of animal substances. It is also used for impressions of the mouth instead of wax, and when it is desirable to have the perfect form of the teeth, it may be superior to wax—as in removing it, the slight elasticity which it possesses, preserves the form of the neck of the tooth; but as a common material to be used for this purpose, we think it inferior to good wax. The degree of heat necessary to make it sufficiently plastic, is too great to be borne in the mouth by many persons.

A solution of this article in Chloroform, forms a useful cement to place between the artificial crown and root of a tooth when about to insert it on a pivot. It also answers for uniting wounds by first intention, and may be used in the same way as the gun-cotton varnish.

Gutta Percha has also been considerably used for filling carious teeth, and as a substitute for amalgam and other cements. We have tried it in various ways, but time is required to test its virtues. The Dentists who have most distinguished themselves for their opposition to amalgam, are unwilling yet to recommend it for this purpose. We shall have more to say upon it hereafter.

